# Supplementary material for: Efficacy and Safety of Transcranial Direct Current Stimulation on Post-Stroke Dysphagia: A Systematic Review and Meta-Analysis
Source: J Clin Med. 2022 Apr 20;11(9):2297. doi: 10.3390/jcm11092297 (PMC9102865; doi:10.3390/jcm11092297)
Supplement: Supplementary file 1 [file jcm-11-02297-s001.zip › jcm-1572811-supplementary.pdf]

Table S1. Search strategy in PubMed.

Order	Search Items
#1	MeSH: "dysphagia"
#2	Ti/Ab: "deglutition" OR "swallowing" OR "dysphagia"
#3	#1 AND #2
#4	MeSH: "stroke"
#5	Ti/Ab: "stroke" OR "cerebral hemorrhage" OR "cerebral infarction" OR "cerebrovascular accident" OR "brain vascular accident"
#6	#4 OR #5
#7	Ti/Ab: "transcranial direct current stimulation" OR "tDCS"
#8	#3 AND #6 and #7

Abbreviation: [Title/Abstract]: Ti/Ab.

Table S2. Search strategy in others database *.

Order	Search Items
#1	Ti/Ab/keywords: "deglutition" OR "swallowing" OR "dysphagia"
#2	Ti/Ab/keywords: "stroke" OR "cerebral hemorrhage" OR "cerebral infarction" OR "cerebrovascular accident" OR "brain vascular accident"
#3	Ti/Ab/keywords: "transcranial direct current stimulation" OR "tDCS"
#4	#1 AND #2 and #3

* Cochrane Library, Web of Science, SinoMed, and VIP; Abbreviation: [Title/Abstract]: Ti/Ab.

Table S3. Search strategy in others database **.

Order	Search Items
#1	Ti/Ab/keyword/free-text terms/ subject words: "deglutition" OR "swallowing" OR "dysphagia"
#2	Ti/Ab/keyword/free-text terms/subject words: "stroke" OR "cerebral hemorrhage" OR "cerebral infarction" OR "cerebrovascular accident" OR "brain vascular accident"
#3	Ti/Ab/keyword/free-text terms/subject words: "transcranial direct current stimulation" OR "tDCS"
#4	#1 AND #2 and #3

**CNKI, Wan-fang database; Abbreviation: [Title/Abstract]: Ti/Ab.
